# Supplementary material for: IgM and IgD B cell receptors differentially respond to endogenous antigens and control B cell fate
Source: eLife. 2018 Mar 9;7:e35074. doi: 10.7554/eLife.35074 (PMC5897097; doi:10.7554/eLife.35074)
Supplement: Transparent reporting form [file elife-35074-transrepform.pdf]

eLIFE
1st Floor
24 Hills Road
Cambridge CB2 1JP, UK
P 01223 855340
W elifesciences.org
T @elife

eLife's transparent reporting form

We encourage authors to provide detailed information *within their submission* to facilitate the interpretation and replication of experiments. Authors can upload supporting documentation to indicate the use of appropriate reporting guidelines for health-related research (see EQUATOR Network), life science research (see the BioSharing Information Resource), or the ARRIVE guidelines for reporting work involving animal research. Where applicable, authors should refer to any relevant reporting standards documents in this form.

If you have any questions, please consult our Journal Policies and/or contact us: editorial@elifesciences.org.

Sample-size estimation

- You should state whether an appropriate sample size was computed when the study was being designed
- You should state the statistical method of sample size computation and any required assumptions
- If no explicit power analysis was used, you should describe how you decided what sample (replicate) size (number) to use

Please outline where this information can be found within the submission (e.g., sections or figure legends), or explain why this information doesn't apply to your submission:

For cell surface markers, GFP reporter expression, and protein phosphorylation, we performed pilot experiments to quantify effect size and phenotype variability. As these parameters generally have standard deviations approximately equal to 10% of their mean and we wanted to detect differences greater than 25%, n = 3 was generally sufficient to demonstrate statistical significance ($p < 0.05$) with at desired power (80%) using a two sample t test. For quantification of rare cell populations (e.g. spontaneous *Lyn*$^{-/-}$ germinal center cells and IgD-only B1a cells) we used n = 5-6 mice because biological variability was higher (SD = 15%). For immunization experiments, biological variability is more pronounced (SD = 30%). In order to detect a difference of immune responses $\geq$ 50% with identical power and significance, we used a sample size of 5-6.
For autoantibody experiments, our previous experience of *Lyn*$^{-/-}$ mice and related genotypes suggested that autoantibody production is only partially penetrant on the B6 genetic background (50% by 6 months of age). Moreover, we previously observed that variation among a group was sizeable (SD = 50%). For this reason, in order to detect a 50% difference in this phenotype with the same power and significance, we studied 11-12 mice per genotype at multiple time points so we could capture at least 16 serum samples per genotype with detectable autoantibody titers. As described in our results section, we collected additional *IgM*$^{+/-}$ *Lyn*$^{-/-}$ samples because this genotype displayed a lower than expected disease penetrance. This enabled us to assess for dissociation of autoantibody production between two genetic loci.

Power and sample size calculator used was: www.stat.ubc.ca/~rollin/stats/ssize/n2.html (Rollin Brant, University of British Columbia)

Because we often combined analyses of different phenotypes into the same experiment, actual sample size may be higher than the minimum recommended by power analysis.

Replicates

- You should report how often each experiment was performed
- You should include a definition of biological versus technical replication

- The data obtained should be provided and sufficient information should be provided to indicate the number of independent biological and/or technical replicates
- If you encountered any outliers, you should describe how these were handled
- Criteria for exclusion/inclusion of data should be clearly stated
- High-throughput sequence data should be uploaded before submission, with a private link for reviewers provided (these are available from both GEO and ArrayExpress)

Please outline where this information can be found within the submission (e.g., sections or figure legends), or explain why this information doesn't apply to your submission:

The number of times each experiment was performed can be found in the corresponding figure legend. We performed experiments at least three times wherever we claimed a difference between experimental groups. We performed experiments at least twice when experimental groups showed no differences, or when our experiments replicated published data.

We define biological replicates as independent analyses of cells isolated from different mice of the same genotype, and we define technical replicates as analyses of cells isolated from the same mouse and used in the same experiment. When technical replicates were used, we averaged them to calculate a value for the biological replicate. All reported values and statistics correspond to biological replicates only, and all "n" values reported reflect the number of biological replicates. We outline our definition of technical and biological replicates in our materials and methods section.

One $IgM^{+/-}$ mouse immunized with sheep red blood cells (Figure 7E-F, 7S1C-D) did not display an expanded plasma cell compartment (0.517% of splenocytes vs. 0.400-0.519% range in unimmunized mice) and was excluded from analysis. We define our exclusion criteria in the materials and methods section.

Statistical reporting

- Statistical analysis methods should be described and justified
- Raw data should be presented in figures whenever informative to do so (typically when N per group is less than 10)
- For each experiment, you should identify the statistical tests used, exact values of N, definitions of center, methods of multiple test correction, and dispersion and precision measures (e.g., mean, median, SD, SEM, confidence intervals; and, for the major substantive results, a measure of effect size (e.g., Pearson's r, Cohen's d)
- Report exact p-values wherever possible alongside the summary statistics and 95% confidence intervals. These should be reported for all key questions and not only when the p-value is less than 0.05.

Please outline where this information can be found within the submission (e.g., sections or figure legends), or explain why this information doesn't apply to your submission:

When comparing two groups, we generally employed Welch's t test, which is more stringent than Student's t test and does not assume that the groups have the same standard deviation. We used paired t tests when studying parameters in allotype-heterozygous mice that are sensitive to cell-extrinsic factors (e.g. dose of immunogen or severity of autoimmune disease). When comparing three genotypes (e.g. receptor levels on WT vs. $IgM^{-/-}$ vs. $IgD^{-/-}$), we used one-way ANOVA and Tukey's Multiple Comparison Test to calculate p values. These standards are described in the materials and methods section, and the statistical test used for each figure panel is described at the end of each figure legend.

When phenotypes were especially variable or stochastic (e.g. autoantibody production), we displayed raw data in our figures. In other cases, the SEM was small and displayed as error bars; showing the raw values would visually impede interpretation of complex graphs. We generally report p values with asterisks* to allow for optimal visual interpretation of graphs; in many figure panels, reporting exact p values would require shrinking the graph features and negatively impact readability. The exact p values are contained in uploaded primary prism files.

(For large datasets, or papers with a very large number of statistical tests, you may upload a single table file with tests, Ns, etc., with reference to sections in the manuscript.)

Group allocation

- Indicate how samples were allocated into experimental groups (in the case of clinical studies, please specify allocation to treatment method); if randomization was used, please also state if restricted randomization was applied
- Indicate if masking was used during group allocation, data collection and/or data analysis

Please outline where this information can be found within the submission (e.g., sections or figure legends), or explain why this information doesn't apply to your submission:

As we used inbred mouse strains with genetically identical littermates, randomization was unnecessary.

Additional data files ("source data")

- We encourage you to upload relevant additional data files, such as numerical data that are represented as a graph in a figure, or as a summary table
- Where provided, these should be in the most useful format, and they can be uploaded as "Source data" files linked to a main figure or table

- Include model definition files including the full list of parameters used
- Include code used for data analysis (e.g., R, MatLab)
- Avoid stating that data files are "available upon request"

Please indicate the figures or tables for which source data files have been provided:

Representative histograms were generated from .fcs files that are prohibitively large to upload. We have provided Prism and/or .xlsx files with raw values for all graphs shown in the paper. Python source code is provided for the binning program in Figure 1F.
